# Caught in the crossfire: cardiac complications of cancer therapy

**DOI:** 10.1172/JCI198289

**Published:** 2026-01-16

**Authors:** Giulia Guerra, Marco Mergiotti, Hossein Ardehali, Emilio Hirsch, Alessandra Ghigo

**Affiliations:** 1 Department of Molecular Biotechnology and Health Sciences, Molecular Biotechnology Center “Guido Tarone”, University of Torino, Torino, Italy.; 2 University of Arizona College of Medicine, Tucson, Arizona, USA.

## Abstract

Advances in cancer therapy have greatly extended patient survival but have also introduced a growing burden of cardiovascular toxicity that threatens long-term outcomes. These toxicities encompass a broad and often unpredictable range of clinical presentations, complicating oncologic care. Understanding how chemotherapy, targeted agents, and immune modulators impair cardiovascular function is essential for early detection, prevention, and management. Emerging insights into the cellular and molecular mechanisms, ranging from immune activation to transcriptional reprogramming and disrupted intercellular communication, underscore the complexity of cancer therapy–induced cardiac injury. Unraveling these mechanisms will be key to developing personalized, mechanism-based strategies that preserve cardiac function without compromising anticancer efficacy. As survivorship continues to improve, mitigating cardiotoxicity remains a critical priority for preserving both the quality and duration of life of patients.

## Overview

Cancer is one of the leading causes of death worldwide, driving ongoing research efforts to develop improved therapeutic strategies. The objective of modern oncology is to prevent early cancer development and to control tumor growth, while minimizing damage to healthy tissues and preserving the quality of life of patients with cancer (1). Accordingly, in addition to conventional chemotherapy, new treatment modalities have been developed, including targeted therapies with small molecules, monoclonal antibodies (mAbs), and promising immune modulators (2). Despite their clinical benefits, both conventional chemotherapy and modern targeted or immune therapies are associated with substantive adverse effects, including fatigue, skin reactions, and gastrointestinal and cardiovascular complications (3). Among these, cancer therapy–related cardiovascular toxicity (CTR-CVT) has emerged as a major concern during both treatment and survivorship, encompassing myocardial, vascular, arrhythmic, pericardial, and valvular complications. The growing recognition of CTR-CVT has driven the development of cardio-oncology as a multidisciplinary specialty dedicated to prevention, early detection, and management (4, 5). Current cardio-oncology care spans baseline risk assessment, surveillance during therapy, and survivorship starting 12 months after treatment (5). The 2022 European Society of Cardiology (ESC) Guidelines further advanced the field by introducing a unified framework and standardized definitions for various forms of CTR-CVT, including the most prevalent entity, cancer therapy–related cardiac dysfunction (CTRCD) (4, 5).

How can cancer be treated without compromising the heart? Why do therapies such as anthracyclines, targeted agents, and immune checkpoint inhibitors (ICIs) give rise to distinct forms of cardiac injury? What molecular pathways determine the heart’s vulnerability to treatment-induced stress, and how can these insights be translated into protective strategies? These are among the central questions driving the field of cardio-oncology. Addressing them is essential to understand the mechanisms of cardiotoxicity and to guide the development of safer, more effective interventions. A clearer view of how each class of anticancer agent affects cardiovascular function, particularly in a tissue with limited regenerative capacity, is expected to reveal opportunities for therapeutic refinement. The following sections examine the cardiovascular impact of specific oncological treatments, linking clinical observations with underlying mechanisms to support the future development of effective cardioprotective approaches for cancer patients. To ensure relevance and novelty, original research articles published within the past five years (2020–2025) were prioritized, while selectively incorporating seminal studies from earlier periods to provide essential background and context for emerging findings. Preclinical data were emphasized in mechanistic discussions or when higher-level clinical evidence was unavailable, whereas prospective human studies and randomized clinical trials were prioritized whenever possible.

## Cardiotoxicity of chemotherapies

### Cardiotoxicity of anthracyclines

Anthracyclines are among the most effective chemotherapeutic agents and remain a mainstay in the treatment of various malignancies, including breast cancer, leukemia, lymphoma, and sarcomas. Their mechanism of action involves DNA intercalation, inhibition of topoisomerase II, and generation of reactive oxygen species (ROS), leading to potent antitumor effects (6–8). All anthracyclines carry a well-established risk of dose-dependent cardiotoxicity, particularly above 250–300 mg/m² (9, 10). The risk increases in patients with cardiovascular comorbidities, older age, female sex, or specific genetic variants (9, 11, 12) and is further amplified by cumulative dosing and cotreatment with trastuzumab or mediastinal radiotherapy (13–15).

#### Clinical features and definitions.

**Figure 1:**
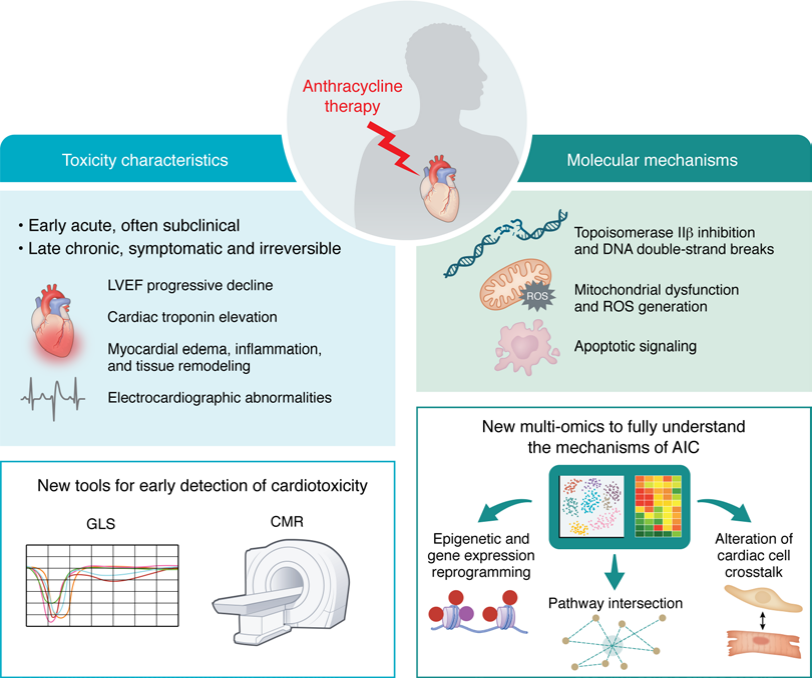
Characteristics and mechanistic landscape of anthracycline-induced cardiotoxicity. Left: AIC often begins as early, subclinical myocardial injury that may progress toward overt chronic dysfunction, typically marked by a decline in left ventricular ejection fraction (LVEF). Elevations in cardiac troponin are frequently observed shortly after anthracycline exposure and reflect early cardiomyocyte injury. Myocardial edema and inflammation have been detected both in patients and in preclinical models, suggesting that inflammatory activation may occur at an early stage. Electrophysiological changes, including QT interval prolongation, point to early disruption of cardiac ion channel function. Advanced noninvasive imaging tools, such as global longitudinal strain (GLS) and cardiac magnetic resonance (CMR), enable detection of subtle myocardial dysfunction before structural deterioration becomes clinically evident. Timely identification through these advanced tools is essential to intervene before irreversible myocardial remodeling takes place. Right: Oxidative stress, mitochondrial damage, and apoptosis are well-established mechanisms of AIC. Multiomic approaches, including single-cell and spatial transcriptomic as well as epigenomic profiling, have begun to reveal complex transcriptional reprograming impacting key pathways of metabolism, inflammation, and structural homeostasis. These approaches also underscore a central role of intercellular crosstalk perturbations in driving long-term toxicity. Together, this emerging framework may support a more integrated understanding of anthracycline cardiotoxicity and guide the development of targeted interventions.

Anthracyclines primarily cause CTRCD, which presents as either symptomatic or asymptomatic left ventricular (LV) impairment (5). Asymptomatic cases are defined by a drop in LV ejection fraction (LVEF) from >10% to <50%, or a >15% reduction in global longitudinal strain (GLS) without symptoms. Symptomatic presentations include clinical signs of heart failure (HF) regardless of LVEF (5). Anthracycline-induced cardiotoxicity (AIC) can manifest acutely within days or weeks of treatment or appear years later as chronic cardiomyopathy, affecting 3%–26% of patients depending on cumulative dose and individual risk factors (16, 17). Clinical signs of AIC often emerge during a subclinical phase, in which myocardial injury progresses silently, and unless identified and managed promptly, may evolve into irreversible dysfunction for which therapeutic options are limited and outcomes poor. Among emerging tools, GLS outperforms traditional LVEF in detecting subtle declines in contractility, identifying early dysfunction in 30.2% of patients compared with 16.4% by LVEF alone (18), while cardiac magnetic resonance (CMR) with T1 and T2 mapping can reveal diffuse inflammation, edema, and fibrosis before overt contractile failure (19–21). In parallel, electrocardiographic abnormalities, such as QT prolongation and arrhythmias, frequently reflect early disruptions in ion channel conductance and autonomic regulation (22) (Figure 1). Importantly, clinical studies suggest that myocardial dysfunction detected at this stage may still be at least partially reversible if identified promptly, whereas delayed detection often coincides with irreversible myocyte loss and fibrosis (14).

#### Mechanisms of cardiotoxicity.

The pathogenesis of AIC is multifactorial and remains incompletely understood. Numerous molecular mechanisms underlying AIC have been extensively characterized and thoroughly reviewed in the literature (23–25). A central mechanism involves the inhibition of topoisomerase IIβ in cardiomyocytes, leading to DNA double-strand breaks and dysregulated gene expression (7, 26). Equally critical is the accumulation of mitochondrial iron, which binds to doxorubicin and catalyzes the generation of ROS via Fenton reactions, resulting in mitochondrial dysfunction and oxidative stress (26). Autophagy inhibition, and the ensuing accumulation of damaged mitochondria, has also been proposed as a key mechanism of AIC (27, 28). Altogether, these events drive cardiomyocyte loss and progressive contractile dysfunction. Recent advances in single-cell transcriptomics, spatial transcriptomics, and epigenomics are reshaping this framework by uncovering previously unrecognized, spatially patterned and cell-type–specific responses (29–32). Transcriptomic studies, in particular, demonstrate that anthracyclines rapidly induce proapoptotic, inflammatory, and stress-responsive gene programs in cardiomyocytes (33), suggesting that early molecular reprogramming may play a key role in determining the trajectory of cardiac injury. CRISPR-based screens have identified genetic determinants of susceptibility, such as drug transporters (ABCC1–ABCC5), redox enzymes (CBR3), and cytoskeletal regulators (SYNPO2) (23). Epigenetic dysregulation is another key feature of AIC. Doxorubicin alters DNA methylation and histone acetylation (e.g., H3K27, H3K4), impacting the expression of genes involved in metabolism, calcium handling, and fibrosis (34, 35). These changes may persist after drug clearance, contributing to a long-term “memory” of injury. Mitochondrial dysfunction exacerbates this process by depleting metabolites, such as acetyl-CoA and NAD+, which are required for chromatin remodeling enzymes (23). Studies using human induced pluripotent stem cell–derived cardiomyocytes (hiPSC-CMs) have shown that interindividual differences in gene expression and splicing following anthracycline exposure strongly correlate with in vitro cell damage and are predictive of in vivo cardiotoxicity risk (36). However, these models are constrained by incomplete cellular maturation, and the majority of transcriptomic and epigenomic signatures identified to date remain exploratory, lacking validation as clinically actionable biomarkers (33).

Increasing evidence indicates that cardiac cell types other than cardiomyocytes play a pivotal role in the pathogenesis of AIC. Anthracycline exposure can impair cardiac progenitor cell survival and paracrine signaling, limiting myocardial regeneration and vascular support and predisposing to maladaptive remodeling (37–39). Indeed, juvenile exposure depletes progenitor pools and heightens vulnerability to later injury in mice (40). Additionally, endothelial dysfunction contributes to vascular damage via activation of the cGAS/STING pathway and downstream interferon signaling, further compromising myocardial integrity (41). Anthracyclines also promote immune cell recruitment by upregulating ICAM-1 on fibroblasts, leading to CD8^+^ T cell–mediated inflammation and fibrosis, a mechanism confirmed in murine models and patient samples (42). B cells also modulate the cardiac response to injury, with FcγRIIB upregulation shown to dampen inflammation (43). Although supported by some human data, these mechanisms remain largely preclinical, with no interventional studies yet confirming their clinical significance.

#### Therapeutic strategies.

Despite advances in understanding the underlying mechanisms, few targeted therapies have been developed to effectively prevent and treat AIC. Current strategies focus on reducing anthracycline exposure and mitigating downstream effects (Table 1). These include dose limitation, prolonged infusions (44), and liposomal formulations (45, 46). Conventional agents, such as ACE inhibitors (47, 48) and beta blockers (49), offer partial cardioprotection, although with inconsistent effects on LVEF. Dexrazoxane is currently the only FDA-approved cardioprotective agent for AIC. While recent reports indicate that its protective mechanism also involves inhibition of Top2β-mediated DNA damage in cardiomyocytes, it was initially described as acting through iron chelation (50, 51). Mechanistically, dexrazoxane reduces mitochondrial iron accumulation, thereby limiting mitochondrial dysfunction and cardiomyocyte death (26). Clinically, dexrazoxane has been shown to lower the risk of HF and preserve LVEF in patients receiving anthracyclines (52). While early concerns about reduced cancer treatment efficacy and secondary cancers have been challenged by recent studies, the topic remains debated due to differing interpretations across patient groups and settings (53–55). Emerging therapies include sodium-glucose cotransporter 2 (SGLT2) inhibitors, which exhibit antiinflammatory and antifibrotic effects in preclinical models (56), and clinical data suggest cardioprotective potential in anthracycline-treated patients, with HF hospitalizations decreased by 51% and HF diagnoses reduced by 71% (57, 58). Statins have also demonstrated promise in reducing LVEF decline (59). Remote ischemic conditioning (RIC), a noninvasive method involving transient limb ischemia, has shown efficacy in animal models (60) and is currently under clinical investigation (61). Despite these efforts, most existing strategies involve repurposed agents with limited efficacy in preventing irreversible cardiomyocyte loss. The development of therapies targeting early injury mechanisms, particularly those revealed by high-resolution multiomic studies, may offer more durable protection. Intervening during the early, potentially reversible phase of cardiotoxicity may prevent the progression to maladaptive remodeling and chronic dysfunction.

### Other chemotherapy toxicities

Although anthracyclines remain the prototypical agents in the study of chemotherapy-induced cardiotoxicity, increasing evidence shows that other widely used drug classes also cause marked cardiovascular injury. These include alkylating agents, antimetabolites, microtubule inhibitors, platinum-based compounds, and certain anticancer antibiotics. Given the differences in their primary molecular targets and mechanisms of action, these agents collectively demonstrate that cardiotoxicity is not limited to a single class (62).

Alkylating agents such as cyclophosphamide are well documented to cause direct cardiovascular damage. At high doses, cyclophosphamide can induce hemorrhagic myocarditis, endothelial dysfunction, and delayed cardiomyopathy. A reported case of cyclophosphamide-induced cardiomyopathy requiring mechanical circulatory support illustrates the severity of this complication. Histopathologic features included interstitial edema, microthrombi, and hemorrhagic necrosis, indicating injury to both endothelial cells and cardiomyocytes (63). Platinum-based compounds such as cisplatin and carboplatin have been associated with increased long-term cardiovascular events. Cisplatin, in particular, has been linked to endothelial dysfunction, autonomic imbalance, and accelerated atherosclerosis. Circulating biomarkers, like von Willebrand factor and endothelin-1, reflect persistent vascular injury, while mitochondrial dysfunction and oxidative stress contribute to subclinical myocardial strain and eventual HF (64, 65).

Antimetabolites, including 5-fluorouracil (5-FU), are known to cause vasospastic angina and myocardial ischemia, even in patients without preexisting coronary disease. Coronary vasospasm and endothelial dysfunction are thought to underlie this toxicity, which can occur at low doses and is often unpredictable based on traditional risk factors (66, 67). Microtubule inhibitors, such as paclitaxel and docetaxel, are frequently used in combination with anthracyclines and have been associated with bradycardia, conduction abnormalities, and hypotension. These effects may result from histamine release or direct interference with cardiac conduction. Coadministration with anthracyclines appears to increase myocardial vulnerability, although further investigation is needed (55). Proteasome inhibitors used in hematologic malignancies, particularly carfilzomib, have also been linked to cardiovascular toxicity, including HF, hypertension, arrhythmias, and ischemia (68).

Current understanding of nonanthracycline cardiotoxicity largely stems from retrospective analyses and isolated case reports. The absence of standardized definitions and prospective data limits the ability to anticipate or prevent these effects. Dedicated mechanistic studies and clinical research are needed to improve early recognition and guide effective cardioprotective strategies.

## Cardiovascular toxicity of targeted cancer therapies

Targeted therapies have transformed oncology by selectively disrupting tumor signaling or modulating immune activity to enhance anticancer responses. Although designed to spare healthy tissues, they are increasingly linked to cardiovascular toxicities that differ in nature and severity depending on their mechanism of action (5, 69, 70). Recognizing these effects is essential to optimize treatment safety and long-term outcomes. Cardiovascular damage arises through distinct, drug-dependent pathways, with targeted agents broadly classified as small-molecule inhibitors, mAbs, and immunotherapies, such as ICIs and CAR-T cell therapies (69, 71).

### Cardiovascular toxicity of small molecules

**Figure 2:**
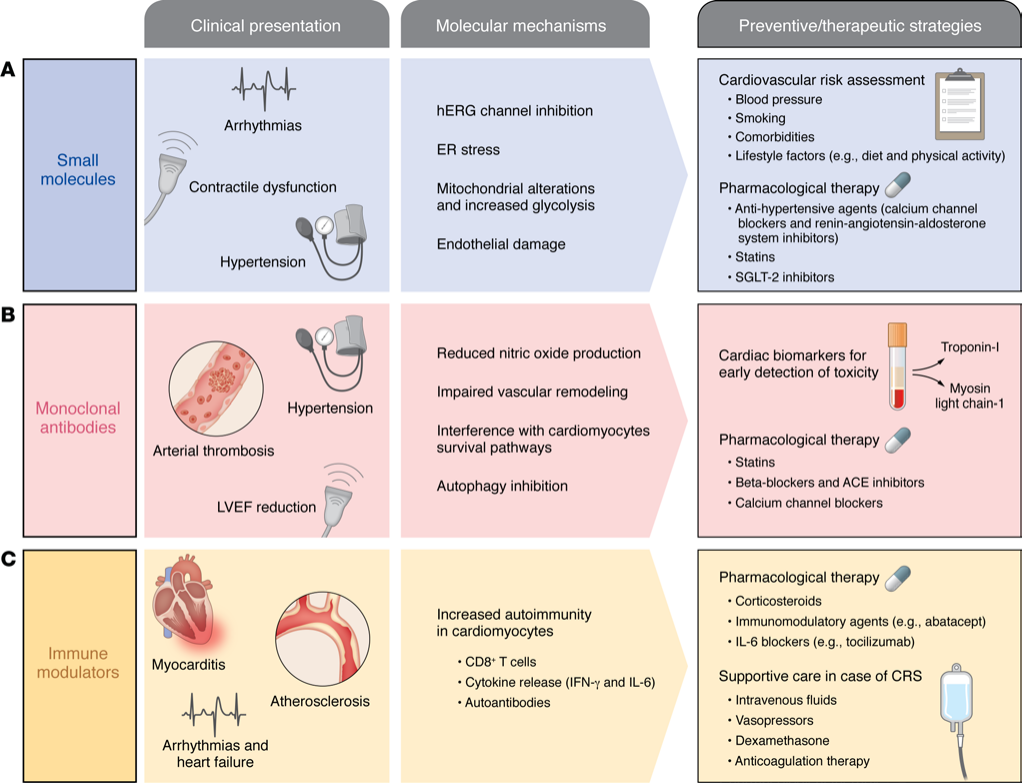
Mechanisms, clinical manifestations, and preventive/therapeutic strategies for cardiovascular toxicity associated with targeted cancer therapies. Targeted cancer therapies can induce cardiovascular toxicities through distinct molecular mechanisms, leading to characteristic clinical presentations and requiring specific management strategies. (**A**) Small-molecule inhibitors cause cardiotoxicity via hERG channel inhibition, mitochondrial dysfunction with increased glycolysis, endoplasmic reticulum stress, and endothelial damage, manifesting as hypertension, arrhythmias, and contractile dysfunction. Management includes cardiovascular risk assessment, lifestyle modifications, and pharmacologic therapies, such as antihypertensive agents, statins, and SGLT2 inhibitors. (**B**) Monoclonal antibodies targeting receptors like VEGF and HER2 impair nitric oxide production, induce vascular remodeling, and alter cardiomyocyte survival signaling, causing hypertension, arterial thrombosis, and reductions in left ventricular ejection fraction (LVEF). Management strategies involve early detection using cardiac biomarkers (e.g., troponin I, myosin light chain-1) and pharmacologic therapy with statins, beta blockers, ACE inhibitors, and calcium channel blockers. (**C**) Immune modulators, including immune checkpoint inhibitors and adoptive cell therapies, increase autoimmunity in the heart through CD8^+^ T cell infiltration, cytokine release (e.g., IFN-γ, IL-6), and autoantibody production, leading to myocarditis, accelerated atherosclerosis, arrhythmias, and heart failure. Management focuses on corticosteroids, immunomodulatory agents (e.g., abatacept, tocilizumab), and supportive care with intravenous fluids, vasopressors, dexamethasone, and anticoagulation in cases of cytokine release syndrome (CRS). This integrated approach highlights the importance of understanding distinct pathogenic mechanisms to guide timely monitoring, prevention, and treatment of cardiovascular changes while maintaining oncologic efficacy.

Cardiovascular toxicity related to small-molecule targeted therapies depends on drug-specific features and the molecular pathways involved (70) (Figure 2), and may be exacerbated when used in combination with other treatments (62, 72). These low–molecular weight compounds interfere with cell surface receptors or intracellular enzymes, such as EGFR, VEGFR, MEK, BCL-2, and BCR-ABL (69), but despite their intended specificity, they can greatly impact cardiovascular health.

Tyrosine kinase inhibitors (TKIs), widely used in cancers such as lung, renal, and melanoma (73, 74), are frequently associated with adverse cardiovascular events including hypertension, arrhythmias, and HF (75–78). Osimertinib, an EGFR-targeting TKI, has been associated with myocardial infarction, LVEF reduction, and valvular dysfunction in approximately 5% of patients (79–81). FDA Adverse Events Reporting System (FAERS) data further indicate increased risks of atrial fibrillation and HF (82), while case reports describe partial LVEF recovery upon treatment discontinuation (83). Ponatinib, used in BCR-ABL–positive leukemias, is associated with thrombocytopenia, arrhythmias, and hypertension (84), while sunitinib has shown marked effects on LVEF and blood pressure in patients (85). A recent prospective study of 78 patients receiving VEGF inhibitors found that 19% developed CTR-CVT, typically within 4 weeks, characterized by a 4.2% LVEF decline and NT-proBNP elevation. Hypertension occurred in 77% of patients, with blood pressure increases after 1 week, underscoring the importance of early monitoring (86).

These toxicities result from on- and off-target effects involving multiple cardiac cell types. TKIs can inhibit hERG channels, causing QT prolongation and arrhythmias in hiPSC-CMs (87, 88), or induce ER stress and mitochondrial damage, impairing contractility (89–91). They also impair cardiac progenitor cell function, reducing reparative capacity (92).

VEGF inhibition disrupts metabolism, endothelial function, and angiogenesis, induces hypertension (93–95), and causes mitochondrial damage with a shift toward glycolysis (85). Hence, [^18^F]-FDG PET may enable early detection (96). These mechanisms are mainly supported by preclinical evidence, and further studies, including randomized trials and meta-analyses, are needed to confirm their clinical relevance. In addition, management strategies should align with the underlying mechanism of toxicity.

Cardiovascular risk assessment helps identify vulnerable patients (97), while vascular dysfunction and cardiac remodeling after administration of the VEGF TKIs necessitate the use of antihypertensive agents, such as calcium channel blockers and potassium-sparing diuretics, which meaningfully reduce blood pressure during therapy with axitinib in a retrospective study (98). Moreover, pretreatment with angiotensin receptor blockers (ARBs), ACE inhibitors, or statins may further attenuate VEGF-TKI–induced hypertension and reduce the risk of cardiovascular events, as reported from patient cohort analyses (99, 100). By preserving mitochondrial function and metabolic balance, SGLT2 inhibitors offer emerging cardioprotective potential in patients with heart dysfunction, with added anticancer effects, highlighting their role in the multidisciplinary care approach (101–103). However, while SGLT2 inhibitors appear to be promising candidates for preventing or treating CTR-CVT from different agents, most available retrospective evidence is derived from diabetic populations, and no prospective randomized controlled trials have yet assessed their efficacy in the CTR-CVT setting. Future studies are therefore essential to overcome these limitations and establish their role in cardio-oncology (104).

### Cardiovascular toxicity of receptor-targeted mAbs

mAbs that target tumor-associated receptors, such as HER2 and VEGF, have become essential in the treatment of several solid tumors. Although generally considered to have a more favorable safety profile compared with traditional chemotherapy, receptor-targeted mAbs can exert clinically meaningful cardiovascular effects (Figure 2). Bevacizumab, which inhibits VEGF signaling, is widely used in breast, colorectal, and other solid tumors. Its cardiovascular toxicity is well documented and includes hypertension, arterial thrombosis, and HF. In a large cohort treated with bevacizumab and trastuzumab, 9.8% experienced major cardiovascular events, including thrombotic events, arrhythmias, and HF (105). A meta-analysis of 77 phase III trials involving VEGF pathway inhibitors (both small molecules and mAbs) reported hypertension, arterial thromboembolism, and cardiac ischemia (106). The pathophysiology involves disruption of endothelial homeostasis, reduced nitric oxide production, and impaired vascular remodeling, which collectively promote vascular stiffness and contribute to cardiac dysfunction (107).

HER2-targeted therapies are a major class of mAbs with cardiac effects. Trastuzumab, the prototype, has shown substantial benefit in HER2-positive breast cancer and led to newer agents such as trastuzumab deruxtecan (DS-8201) and T-DM1 (108–113). Cardiotoxicity typically involves LVEF decline and, in severe cases, HF. Although usually reversible and not dose dependent, it remains clinically relevant, with LVEF decline in 7.5% and HF in 1.9% of patients (114), and risk increases with comorbidities or anthracycline use (115, 116). Mechanistically, preclinical models show that HER2 blockade interferes with cardiomyocyte survival signaling via the PI3K/Akt and ERK/MAPK pathways, impairs mitochondrial function in rat cardiomyocytes, and suppresses autophagy via mTOR, contributing to oxidative stress and contractile dysfunction in human primary cardiac cells (117–121). These effects are partly reversible with AMPK activation, as trastuzumab-treated hiPSC-CMs show recovery of metabolic and contractile function when AMPK is stimulated (122, 123). Emerging evidence suggests that trastuzumab-induced cardiovascular toxicity is not restricted to cardiomyocytes but arises from the interplay of multiple cardiac cell types. In endothelial cells, trastuzumab activates the EGFR/STAT3 pathway and disrupts calcium signaling with cardiomyocytes, thereby aggravating contractile dysfunction in hiPSC-CMs and human-derived cell lines (124). In parallel, studies in mouse models demonstrate enhanced proinflammatory signaling, including TNF-α and TGF-β activation, which is accompanied by immune cell infiltration and fibrosis, further amplifying tissue injury (125, 126). Extending this multicellular perspective, Barth et al. showed that trastuzumab impairs the cardiomyogenic and angiogenic functions of human resident cardiac stem cells in vitro, abolishing their regenerative capacity in mouse models and implicating stem cell dysfunction as an additional pathogenic mechanism (127). Together, these findings outline a multicellular pathogenic network involving endothelial, immune, and stem cells, although clinical validation is still needed to translate these insights into multidisciplinary therapeutic strategies.

Antibody-drug conjugates, such as T-DM1 and DS-8201, appear to be better tolerated from a cardiovascular standpoint, with LVEF reductions in fewer than 1% of patients and only isolated reports of ischemic events (112, 128). However, a recent retrospective analysis of the FAERS database reported an overall incidence of adverse events of 17.12% upon antibody-drug conjugate therapy, with different cardiovascular toxicity from different types of conjugates (e.g., agents targeting HER2 displayed fewer adverse events), highlighting the importance of novel preventive and therapeutic strategies to manage the cardiovascular sequelae of these therapies (129).

Cardioprotective strategies are increasingly integrated into treatment planning for patients receiving HER2- or VEGF-targeted mAbs. Systematic reviews and meta-analyses demonstrate that statins display antiinflammatory and antioxidant properties that may reduce the incidence of CTR-CVT by more than 50%, while beta blockers and ACE inhibitors support continued therapy by mitigating LV dysfunction and remodeling (130). Moreover, antihypertensive agents, such as amlodipine, can be useful in managing bevacizumab-induced hypertension, as indicated from expert recommendations (131). Overall, as the use of receptor-targeted mAbs continues to expand, integrating cardiovascular monitoring and preventive therapies remains essential for maintaining oncologic efficacy without compromising cardiac health.

### Cardiovascular toxicity of immune modulators

ICIs, adoptive cell therapies (ACTs), and cancer vaccines have revolutionized cancer treatment by enhancing immune cell–mediated antitumor responses. ICIs, such as ipilimumab (anti–CTLA-4), nivolumab (anti–PD-1), pembrolizumab (anti–PD-1), and atezolizumab (anti–PD-L1), are compounds that restore T cell activity by blocking inhibitory signals that normally maintain peripheral tolerance (132, 133). ACT is a personalized form of immunotherapy that involves the ex vivo expansion or genetic modification of autologous lymphocytes to enhance antitumor activity. Several ACT strategies have been developed: CAR-T cell therapy, which involves engineering T cells to express CARs targeting antigens such as CD19 or B cell maturation antigen (BCMA); tumor-infiltrating lymphocytes (TILs), which are isolated from patient tumors and expanded ex vivo; bispecific T cell engagers (BiTEs), which are synthetic molecules that simultaneously bind tumor antigens and T cells to promote cytotoxic engagement; and T cell receptor–engineered T (TCR-T) cells, which are modified to express tumor-specific TCRs. Additionally, cancer vaccines aim to train the immune system to recognize and eliminate tumor cells by presenting tumor-associated antigens in combination with immune-stimulating adjuvants, fostering long-term immunological memory and durable tumor control (134, 135).

Despite the high specificity of immune-modulating therapies, these same mechanisms can disturb cardiovascular immune homeostasis (Figure 2). In the myocardium, PD-1/PD-L1 and CTLA-4 pathways protect against CD8^+^ T cell–mediated injury by limiting inflammation and autoimmunity, as demonstrated in animal studies (136–138). Preclinical evidence showed that their inhibition can lead to infiltration by macrophages and cytotoxic CD8^+^ T cells, cytokine release (including IFN-γ and IL-6), and in some cases, autoantibody production against cardiac proteins such as troponin I (139, 140). Moreover, in a mouse model of ICI-related cardiac injury, the involvement of activated myofibroblasts was uncovered, with myofibroblast-derived angiopoietin-like protein 2 (ANGPTL2) as a key mediator that promotes chemokine production and T cell recruitment, thereby exacerbating ICI-related autoimmune myocarditis (141).

In ACTs, particularly CAR-T cell therapy, cardiovascular toxicity is frequently linked to cytokine release syndrome (CRS), a systemic inflammatory condition driven by massive cytokine surges that disrupt vascular and myocardial stability (142, 143). In CRS, IL-6, IFN-γ, and TNF-α drive systemic inflammation, endothelial dysfunction, vascular leak, and myocardial impairment, supported by animal and observational studies (143). Endothelial cells amplify these effects, promoting cytokine storms, vascular leakage, coagulopathy, and blood-brain barrier disruption in CAR-T–treated patients (144, 145). Cardiovascular complications are less documented with BiTE and TIL therapies, but CRS likely plays a key role. Direct cardiotoxicity may also occur through T cell recognition of shared antigens (e.g., titin) or indirect immune-mediated injury via antigen mimicry or alloreactivity, observed in preclinical models and patients (146), although further studies are needed to better define the mechanisms underlying these cardiovascular manifestations.

Clinically, acute CTR-CVT of ICIs are uncommon but can be severe (Table 2). Myocarditis, pericarditis, arrhythmias, and HF have been documented, with myocarditis accounting for up to 25% of ICI-related deaths (147). In a cohort of patients with lung cancer treated with ICIs, 13.3% experienced major cardiac events, especially when combined with VEGF inhibitors (148). Smaller studies have reported LVEF reduction, Takotsubo-like syndromes, and both tachy- and bradyarrhythmias (149–151). A systematic review and meta-analysis of animal studies showed that immune checkpoint inhibition increases atherosclerotic plaque size by more than 50% and T cell/macrophage infiltration (152). Mechanistically, preclinical mouse models demonstrated that PD-1 blockade accelerates plaque inflammation through IFN-γ–driven activation of CCR2^+^ macrophages, a process detectable by PET imaging (153). These experimental findings align with clinical data showing that ICIs promote or worsen atherosclerosis by sustaining chronic T cell activation and vascular inflammation (154), with meta-analyses of randomized trials reporting increased risks of dyslipidemia and myocardial infarction (155). Consistently, ICIs targeting PD-1, PD-L1, and CTLA-4 accelerate plaque progression and heighten the risk of myocardial infarction and stroke by modulating key atheroprotective pathways (156). Highlighting the multidimensionality of ICI-related cardiovascular damage, a myocarditis severity score based on clinical predictors (e.g., troponin elevation, thymoma, reduced LVEF) has shown strong prognostic value and can identify low-risk patients in prospective validation within a multidisciplinary cardio-oncology approach (157).

CAR-T–induced cardiovascular toxicity commonly includes reductions in LVEF, hypotension, and arrhythmias (158, 159). In the CARdio-Tox study, 59.3% of patients experienced cardiac complications, such as LVEF impairment and biomarker elevation, within seven days of therapy (160). These events are closely linked to CRS, which typically causes sinus tachycardia and hypotension but can also lead to ventricular arrhythmias and atrial fibrillation (161). Consequently, CAR-T administration requires intensive monitoring to enable rapid detection and management of complications (162). Cardiovascular toxicity associated with BiTE and TIL therapies appears less pronounced but remains poorly characterized (163). In the TOWER trial of the BiTE agent blinatumomab, grade 3 CRS occurred in 4.9% and acute coronary syndromes in 0.4% of patients (164), while real-world data indicate higher CRS rates (~9%) (165). FAERS reports have also linked BiTE agents to myocarditis, shock, and disseminated intravascular coagulation, often independent of CRS (166). For TIL therapies, reported cardiovascular events include hypotension (32.6%), atrial fibrillation (14%), and troponin elevation (2.3%), without documented cases of LV dysfunction or overt HF (167). Overall, evidence regarding BiTE, TIL, and TCR-engineered T cells remains limited, underscoring the need for systematic surveillance to better define their cardiac toxicity profiles (163) (Table 2). Cancer vaccines, such as those based on mRNA, show strong potential for eliciting targeted antitumor immunity, but their cardiovascular safety remains incompletely understood, particularly in patients with cancer and existing comorbidities (168). The risk of immune-related cardiovascular effects, including myocarditis observed with COVID-19 mRNA vaccines (169, 170), raises concerns given the potent immune activation these therapies can induce and highlights the importance of continuous patient monitoring.

Management of cardiovascular toxicity from immune modulators focuses on immune responses and supportive care. For ICI-induced myocarditis, high-dose corticosteroids remain first-line therapy. In refractory cases, immunomodulatory agents such as abatacept, a CTLA-4–Ig fusion protein, have shown efficacy in reversing immune-mediated cardiac injury in patients (171), while targeting the NLRP3 inflammasome is also being explored in preclinical models of melanoma-bearing mice treated with ICIs (172). In the clinical setting of CAR-T–associated CRS, early administration of tocilizumab, an IL-6 receptor blocker, reduces cardiovascular complications, especially when given within 12 hours of onset (173). Adjunctive therapies may include intravenous fluids, vasopressors, and selective anticoagulation in patients at risk for thrombotic events (163, 174). Continuous cardiac monitoring remains essential for high-risk patients undergoing immunotherapy.

## Cardiovascular complications of combined cancer treatments

Combining anticancer therapies with different mechanisms can enhance tumor control (175, 176) but also increases cardiovascular toxicity by disrupting complementary cardiac pathways. A key example is the combination of anthracyclines and HER2-targeted agents. In HER2-positive breast cancer, anthracyclines followed by trastuzumab increased LVEF decline risk, with 25% developing asymptomatic dysfunction five years after treatment (115, 177). In a retrospective study of 549 patients, 12.5% showed LVEF reduction, strongly associated with preexisting cardiovascular risk factors and concurrent anthracycline and trastuzumab use; partial recovery occurred in 44% but was unpredictable (178). Moreover, long-term follow-up showed that 25% of breast cancer survivors developed asymptomatic systolic dysfunction more than five years after sequential therapy, emphasizing the need for extended monitoring and coordinated cardio-oncology care (115). Concerning the concurrent or sequential administration of anthracyclines and trastuzumab, real-world data confirmed a higher incidence of LVEF decline with concomitant anthracycline and trastuzumab treatment compared with anthracyclines alone (36% vs. 9.5%) (178). In contrast, a randomized trial found no significant difference in CTRCD rates between sequential and concurrent administration (19.4% vs. 22.4%), but the concurrent approach shortened treatment duration without compromising safety (179).

Mechanistically, the combination of anthracyclines with trastuzumab sustains ROS accumulation, amplifies mitochondrial and endoplasmic reticulum stress, and compromises antioxidant defenses, leading to a synergistic and often irreversible pattern of cardiotoxicity (180). In line with this, trastuzumab has been shown to potentiate doxorubicin-induced apoptosis of cardiomyocytes and systolic dysfunction through coactivation of the NLRP3 inflammasome. As a result, elevated ROS and proinflammatory cytokine release were detected both in vitro in primary rat cardiomyocytes, and in vivo, with inhibition of NLRP3 attenuating these toxic effects (181). Accordingly, intravenous delivery of cardiac progenitor cell–derived exosomes has been shown to protect against sequential doxorubicin/trastuzumab-induced cardiotoxicity in a rat model, by attenuating oxidative stress, fibrosis, inflammation, and LV dysfunction through mechanisms involving exosomal miR-146a-5p (182).

While these mechanistic insights stem largely from experimental models, clinical data increasingly confirm the relevance of cardiovascular risk from this combined anticancer approach. Patients receiving anthracyclines and trastuzumab represent a high-risk group requiring intensive cardiovascular surveillance. Current recommendations include baseline echocardiography followed by regular reassessments during and after therapy, with different timing strategies (183, 184). Advanced techniques such as 3D LVEF, strain imaging, and biomarkers (high-sensitivity troponin and NT-proBNP) improve early detection of subclinical dysfunction (184). The CATS study showed that early LVEF decline and lower baseline function independently predict later dysfunction (177), supporting individualized monitoring. Ongoing cardiology involvement remains essential even after treatment completion, as new cardiovascular risk factors frequently emerge during survivorship, especially in anthracycline-trastuzumab–treated patients (5, 115). Regular imaging and biomarker assessments should continue even in patients with preserved LVEF.

Beyond HER2-targeted therapy, combining ICIs with anthracyclines raises concerns about additive cardiotoxicity. Preclinical studies suggest anthracyclines modulate PD-1/PD-L1 signaling, potentially increasing susceptibility to immune-related events during subsequent ICI therapy (185), warranting clinical validation. Another high-risk combination is dual-ICI therapy, which may intensify immune-mediated cardiovascular injury by enhancing T cell activation and promoting off-target infiltration with shared clonal T cell populations in tumor and cardiac tissue. These effects were observed in a case report of two patients with fulminant myocarditis after treatment with ipilimumab and nivolumab (149). Supporting this, VigiBase data show higher risks of myocarditis and fatal cardiac events with ICI combinations versus monotherapy (186), and observational studies report increased myocardial and pericardial toxicity with combination regimens (187). For ICI combinations, guidelines recommend baseline ECG, troponin, and natriuretic peptide testing prior to therapy (188, 189), with repeat assessments before each infusion in patients on combination regimens (189, 190).

Overall, preclinical and clinical evidence suggests that the simultaneous disruption of oxidative stress responses, survival signaling, and immune regulation renders combination therapies particularly hazardous for patients with baseline cardiovascular risk. More data are needed to refine risk quantification and identify vulnerable populations. Clinically, managing CTR-CVT associated with combination regimens requires proactive, long-term strategies emphasizing thorough baseline assessment and continuous monitoring. Risk stratification tools, such as those recommended by the ESC, provide a framework to tailor surveillance and prevention (5). Ultimately, effective management should rely on structured multidisciplinary cardio-oncology care to align cancer treatment goals with cardiovascular protection throughout the continuum of care.

## Future perspectives

The increasing use of cardiotoxic agents in oncology, along with rising cancer survivorship, underscores the need to integrate cardiovascular protection into every phase of cancer care. However, a major translational challenge in contemporary cardio-oncology lies in the heterogeneity and unpredictability of therapy-induced cardiac complications. At present, no consensus risk score reliably combines biomarkers, genetic susceptibility, and clinical characteristics (beyond traditional cardiovascular comorbidities) to accurately stratify individual risk. This lack of robust predictive tools hampers the early identification of vulnerable patients and limits the precision of surveillance and preventive strategies. Consequently, developing validated risk models and novel biomarkers represents a key research priority for enabling personalized assessments of cardiotoxicity (191). In this context, a unified perspective on shared risk factors, mechanisms, and preventive strategies across different anticancer modalities is essential to highlight both the common pathogenic pathways and therapy-specific vulnerabilities that underpin CTR-CVT (Table 3).

**Figure 3:**
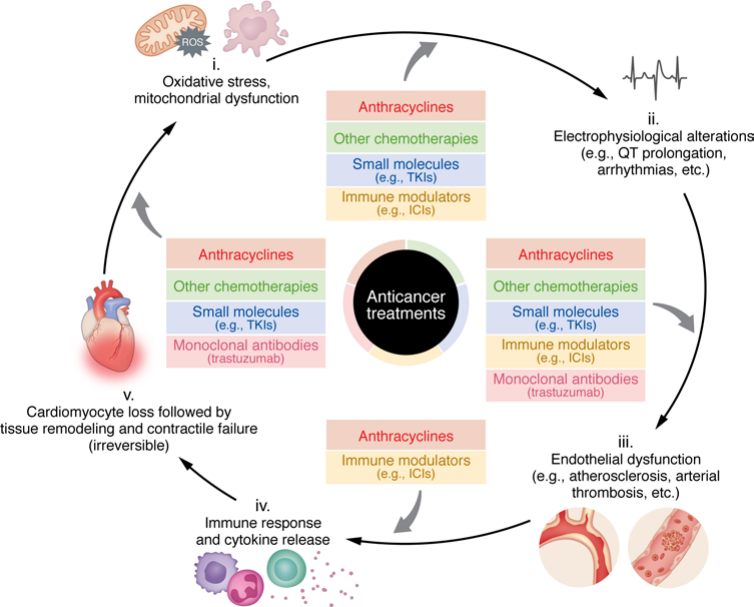
Cancer therapies initiate a vicious cycle of cardiac damage. Different classes of anticancer treatments, including anthracyclines, other chemotherapies, small-molecule inhibitors, monoclonal antibodies, and immune checkpoint inhibitors, converge to initiate interconnected mechanisms that drive cardiotoxicity. Early oxidative stress and mitochondrial dysfunction disturb metabolic homeostasis and calcium handling, leading to redox-sensitive ion channel changes and electrophysiological abnormalities such as QT prolongation and arrhythmias. Persistent injury promotes endothelial dysfunction, with loss of nitric oxide signaling, vascular inflammation, and microvascular rarefaction. These processes enhance immune activation and cytokine release, ultimately resulting in cardiomyocyte loss, fibrotic remodeling, and progressive contractile failure. Each step in the cascade may be differentially engaged by specific therapy classes (entry arrows), yet together they form a shared pathogenic framework. The diagram also conveys a temporal gradient in which upstream events (steps i–iv) are potentially reversible, while chronic structural remodeling (step v) becomes increasingly irreversible.

While major strides have been made in recognizing and classifying treatment-related cardiovascular dysfunction, a deeper understanding of the early events that initiate injury is essential. Oxidative stress, mitochondrial dysfunction, inflammation, and fibrosis can be triggered soon after exposure to cardiotoxic therapies. Early events such as mitochondrial and metabolic stress, ionic imbalance, electrophysiological changes, and inflammatory edema likely mark stages where cardiac injury may still be reversible. If not intercepted, these processes trigger self-perpetuating loops in which oxidative stress, inflammation, and microvascular dysfunction reinforce one another, ultimately leading to cardiomyocyte loss, fibrotic matrix deposition, and persistent structural remodeling with poor clinical recovery (Figure 3). Interrupting these cycles at an early stage may prove more effective than attempting to reverse advanced, chronic damage. Defining the molecular determinants that govern the transition from reversible injury to irreversible dysfunction is therefore a high priority for future research.

Technological advances now make it possible to dissect the pathophysiology of CTR-CVT at unprecedented resolution. Single-cell RNA sequencing and spatial transcriptomics can illuminate cell-type–specific stress responses and infer cell-cell communication networks that occur during the earliest phases of cardiac injury. Combined with epigenomic and proteomic analyses, these approaches are expected to reveal new biomarkers of subclinical damage and identify targets for timely therapeutic intervention. This must necessarily involve the development of appropriate preclinical models across different species that incorporate key aspects of cardiovascular toxicity, some of which have been overlooked so far, such as interorgan interactions, genetic predisposition, sex-specific risks, and racial or ethnic disparities (192). Sex and ethnicity also meaningfully influence biomarker levels and cardiotoxicity risk in cardio-oncology. Cardiac troponin baseline levels are consistently lower in women due to smaller myocardial mass and hormonal influences, leading to underestimation of cardiotoxicity in women receiving anthracyclines or trastuzumab (5). Similarly, genetic markers like *RARG* variants and *NAD(P)H* oxidase polymorphisms linked to anthracycline cardiotoxicity show different allele frequencies by ancestry, influencing susceptibility (193, 194). Yet, existing cardiotoxicity risk models do not incorporate sex or ethnicity explicitly, despite evidence of differential predictive performance.

Another important translational challenge is the need to better align preclinical cardio-oncology research with the evolving landscape of clinical oncology (195). Current evidence highlights a disproportionate emphasis on anthracycline-induced cardiovascular toxicity in preclinical studies, despite its decreasing prevalence in contemporary clinical practice. Hence, preclinical research in the field of cardio-oncology should be refocused on the most clinically relevant toxicities.

Therapeutic development should increasingly focus on mechanism-specific interventions that interrupt maladaptive responses at their inception. These may include antiinflammatory agents, mitochondrial protectants, metabolic modulators, and therapies aimed at preserving endothelial and cardiomyocyte crosstalk. At the same time, the design of next-generation anticancer drugs with reduced cardiotoxic potential remains an essential objective to improve the long-term safety of oncologic care.

Equally important is the translation of emerging concepts, such as cardio-oncology survivorship, long-term remodeling, and preclinical injury, into structured multidisciplinary care models, as recommended by ESC Guidelines (5). Survivorship is an extended phase where late cardiovascular sequelae often appear, requiring longitudinal pathways that integrate oncology and cardiology for ongoing risk assessment, imaging, and biomarker surveillance. Early identification of preclinical injury provides a crucial window for intervention, which can only be fully leveraged through coordinated workflows among imaging specialists, laboratory medicine, cardiologists, and oncologists. Moreover, recognizing long-term remodeling as a dynamic, progressive process underscores the importance of sustained cardio-oncology follow-up embedded within survivorship clinics, enabling timely anticipation and management of evolving cardiovascular complications.

Clinically, the future of cardio-oncology lies in the implementation of personalized, risk-adapted surveillance and intervention strategies. Integration of imaging, circulating biomarkers, and electronic health data into predictive algorithms may enable earlier detection and prevention of cardiac events. Ultimately, a multidisciplinary effort combining insights from molecular biology, clinical cardiology, and oncology will be required to develop therapeutic strategies that sustain both cancer remission and cardiovascular health.

## Funding support

- Italian Ministry of Health (grant GR-2021-12371950 to AG).
- Italian Ministry of Education, Universities and Research (grants PRIN 2022 20223YPL49 and PRIN PNRR 2022 P2022ZB72T to AG; PRIN 2022 PNRR P20229WSC9 to EH).
- Worldwide Cancer Research (grant 25-0373 to AG).
- Leducq Transatlantic Network of Excellence (grant 19CVD02Le to EH and HA).

## Figures and Tables

**Table 2:**
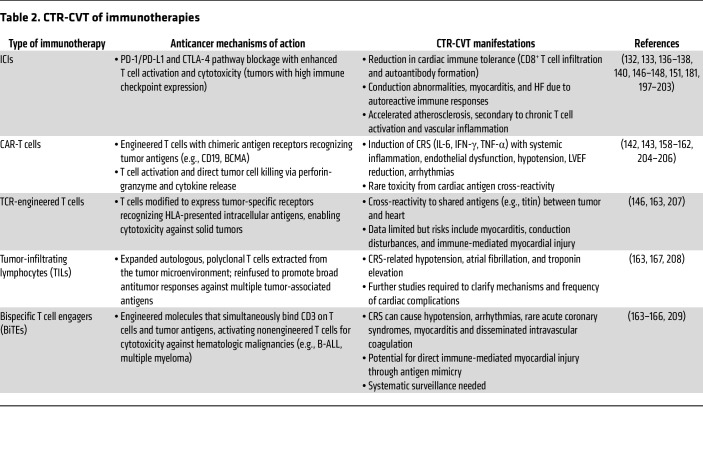
CTR-CVT of immunotherapies

**Table 1:**
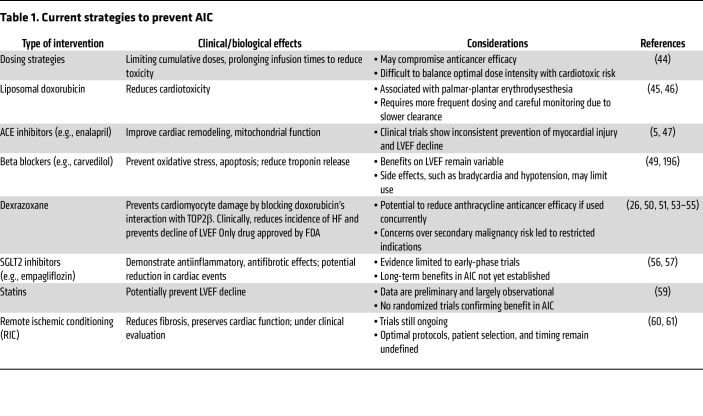
Current strategies to prevent AIC

**Table 3:**
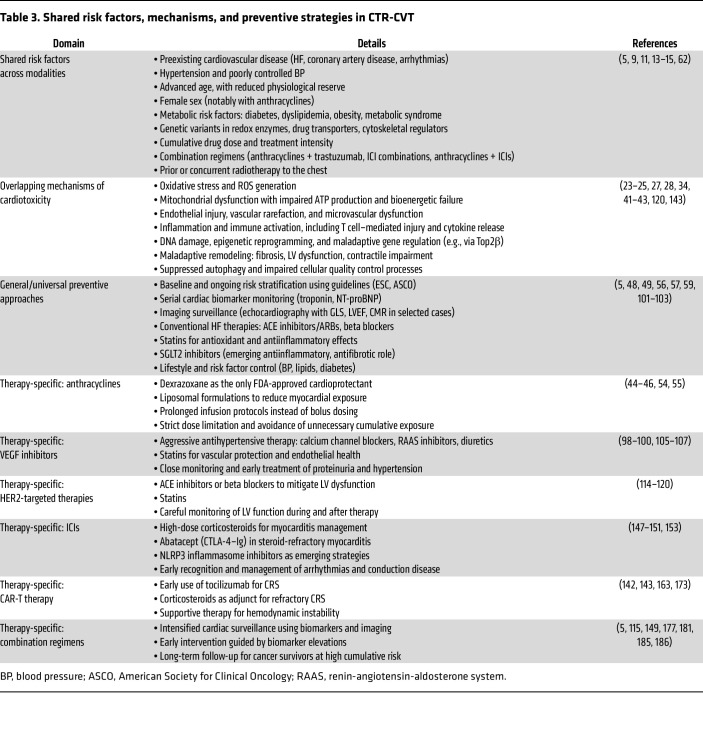
Shared risk factors, mechanisms, and preventive strategies in CTR-CVT

## Version 1 01/16/2026

Electronic publication
